# Rationale and design of PROSPECT-CONKO 004: a prospective, randomized trial of simultaneous pancreatic cancer treatment with enoxaparin and chemotherapy)

**DOI:** 10.1186/1471-2407-8-361

**Published:** 2008-12-05

**Authors:** Hanno Riess, Uwe Pelzer, Andreas Hilbig, Jens Stieler, Bernhard Opitz, Theo Scholten, Dörte Kauschat-Brüning, Peter Bramlage, Bernd Dörken, Helmut Oettle

**Affiliations:** 1 Charité Campus Virchow-Clinic, Medical Clinic Hematology/Oncology, Augustenburger Platz 1, 13353 Berlin, Germany; 2 Hospital St. Elisabeth/St. Barbara Halle, Mauerstraße 5, 06110 Halle (Saale), Germany; 3 Hospital Hagen, Grünstr. 35, 58095 Hagen, Germany; 4 Sanofi-Aventis Deutschland GmbH, Potsdamer Strasse 8, 10785 Berlin, Germany; 5 Institute for Clinical Pharmacology, Medical Faculty, TU Dresden, Fiedlerstrasse 27, 01307 Dresden, Germany

## Abstract

**Background:**

Advanced pancreatic cancer, in addition to its high mortality, is characterized by one of the highest rates of venous thromboembolic events (VTE) as compared to other types of cancer. Enoxaparin, a low molecular weight heparin (LMWH), has proven to be effective for the prevention and treatment of VTE in surgical and general medical patients. Results of some small studies suggest that this benefit might extend to patients with cancer, however, enoxaparin is not currently indicated for this use. This phase IIb study was designed to analyze the efficacy of enoxaparin in patients with locally advanced or metastatic pancreatic cancer undergoing systemic chemotherapy.

**Methods:**

The aim of this prospective multicenter trial is to compare concomitant treatment with enoxaparin to no anticoagulation in 540 patients. Primary endpoint is the incidence of clinically relevant VTE (symptomatic deep venous thrombosis (DVT) of the leg and/or pelvic and/or pulmonary embolism (PE)) within the first 3 months. Secondary endpoints include the incidence of symptomatic and asymptomatic VTE after 6, 9 and 12 months as well as remission at 3, 6, 9 and 12 months, overall survival and bleeding. Trial registration: isrctn.org identifier CCT-NAPN-16752, controlled-trials.com identifier: ISRCTN02140505.

**Results:**

An interim analysis for safety performed after inclusion of 152 patients revealed no increased risk of bleeding (5 pts vs. 6 pts, Chi^2^: 0.763).

**Conclusion:**

PROSPECT is a pivotal study in elucidating the role of low molecular weight heparins in advanced pancreatic cancer. Its results will lead to a new understanding of the role of heparins in the prevention of venous thromboembolism and of their effect on survival, remission rates and toxicity of chemotherapeutic regimens.

## Background

Pancreatic cancer is among the most common malignancies in the world with about 232,000 new cases every year [1]. Due to its aggressive nature this illness accounts for around 32,000 deaths per year in the United States [2], and around 47,000 in Western Europe [3]. The median survival time is 6–10 months with locally advanced disease and 3 to 6 months in patients with metastases. Without any specific anticancer therapy the median overall survival is between 2 to 4 months.

In addition to the poor over-all prognosis the course of the disease is often complicated by thromboembolic events. Lower-extremity deep venous thrombosis, thrombophlebitis migrans, and pulmonary embolism are among the well-known presentations in pancreatic cancer. Further manifestations also include disseminated intravascular coagulation, splenic vein thrombosis, portal or superior mesenteric vein thrombosis, and spontaneous arterial thromboembolism, extremity ischemia, and mesenteric or iliofemoral occlusion [4-7].

**Figure 1:**
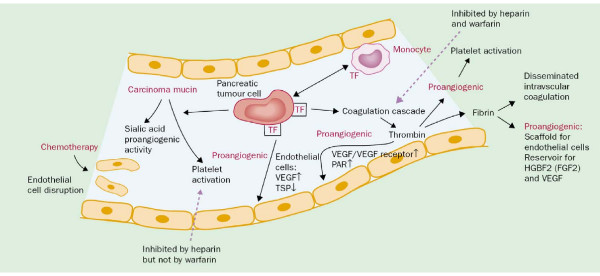
**Initiation of the coagulation and angiogenic cascades by pancreatic tumour cells (modified from **[61]). Figure legend text: TF, tissue factor; VEGF, vascular endothelial growth factor; PAR, protease-activated receptor; TSP, thrombospondin; HGBF2, heparin-binding growth factor 2 (previously called fibroblast growth factor 2 [FGF-2]).

The first report describing the relationship between pancreatic cancer and thrombosis was published in 1938, documenting a 60% prevalence of venous thrombosis in various locations upon autopsy compared with 15–25% in other malignancies [8]. This makes pancreatic cancer the tumour entity with the highest VTE rates. Since then, further studies have confirmed the association of pancreatic adenocarcinoma with VTE reporting prevalence rates of 5 to 60% [9-12]. In a recent cohort study in 202 patients with pancreatic cancer (based on histological and cytological examinations or ultrasound and CT) the incidence of VTE was 108.3 per 1,000 patient-years (10.8%) resulting in a 58.6-fold increase in relative risk as compared with an age- and sex-adjusted general population [13]. Patients treated with chemotherapy have further a 4.8-fold increased risk for VTE [14-16], whereas the risk increase is at worst moderate with radiotherapy [17]. In a study conducted in 7,000 patients with different cancers, Sorensen and colleagues found that 12% of patients with and 36% of patients without VTE were alive at one year [18], a result documenting the negative prognostic implication of clinical VTE in cancer patients. The prothrombogenic effects of malignant disease have been extensively studied and revealed direct and indirect activation of coagulation, inhibition of fibrinolysis, enhancement of adhesion of blood cells and/or the initiation of an inflammatory state [19,20] (Figure 1). For an excellent overview see Nakchbandi and Löhr [21].

Following the initial observation that cancer patients tend to develop venous thrombosis different anticoagulants were tested to decrease the risk of VTE. Three studies showed the superiority of low molecular weight heparins in terms of a favourable benefit risk ratio in this indication [22-24] in comparison to oral anticoagulants. The incidence of bleeding so far described for cancer patients receiving oral anticoagulant treatment for VTE is markedly lower than the high incidence of recurrent venous thrombosis in these cancer patients [25]. In retrospective meta-analyses from randomized trials of VTE patients initially treated with low molecular weight heparin (LMWH) it was demonstrated that mortality after 3 months was up to 50% lower as compared to patients started on anticoagulation with unfractionated heparin [26-29]; this beneficial effect of LMWH on mortality nearly exclusively resulted from the effect observed in cancer patients [26,28].

In addition to anticoagulation inhibiting VTE events there is evidence that LMWH have additional pleitropic effects. The low incidence of malignancies in patients using long-term anticoagulant therapy raised the possibility of antineoplastic activities of these drugs [26,28,30]. Also, dramatic tumour regression in some case reports and small studies has suggested the potential for anti-cancer activity of LMWH [26,28,31-33]. LMWH therefore may be regarded not only as a suitable anticoagulant in cancer patients with VTE but as also anti-cancer or anti-metastatic therapeutic principle.

Recent guidelines of the American College of Chest Physicians (ACCP) recommend the use of LMWH for anticoagulation in patients with cancer-induced thrombosis [34]. The LMWH enoxaparin, based on the ENOXACAN II study [35], is effective for the prolonged primary prevention of thrombosis after surgery for abdominal or pelvic cancer and has been recommended for surgical patients with cancer at high risk by the recently published guidelines of the International Union of Angiology [36].

### Therapeutic implications for patients with pancreatic cancer

Given the high incidence of thromboses effective anticoagulant therapy may be warranted in patients with pancreatic cancer [13]. A recent small phase II trial suggested an improved survival by the addition of LMWH to chemotherapy [33]. Icli and colleagues aimed to assess the efficacy of the addition of LMWH to gemcitabine (GEM) plus cisplatinum (CDDP) combination chemotherapy on survival. 69 consecutive patients with advanced pancreatic cancer were treated with GEM (800 mg/m^2^, day 1, day 8) plus CDDP (35 mg/m^2^, day 1, day 8) every 21 days +/-LMWH (nadroparine calcium, 2,850 IU/day until disease progression). Ten out of 35 patients in the LMWH group and 10 out of 34 patients in the chemotherapy alone group had primary inoperable locally advanced disease and the rest of the patients had metastatic disease. Total response rate was reported to be 58.8% (complete remission 11.7%) for the patients treated with LMWH and 12.1% (no complete remission) for those treated without LMWH (P = 0.0001). The LMWH group had a better median time to progression and survival when compared to control group (7.3 vs. 4.0 months, P = 0.0001; 13.0 vs. 5.5 months, P = 0.0001). The toxicity was similar and acceptable in both groups. The authors concluded that the addition of LMWH to the GEM plus CDDP combination significantly improved the response and survival in patients with advanced pancreatic cancer and the current schedule deserves to be tested in future clinical trials.

### Rationale for PROSPECT

Based on these encouraging trial results of LMWH, the aim of Prospective, Randomized trial Of Simultaneous Pancreatic cancer treatment with Enoxaparin (PROSPECT) is to investigate the efficacy of low molecular weight heparin (enoxaparin) for the prevention of venous thromboembolism in patients with advanced localized or metastasized pancreatic cancer as a primary endpoint.

## Methods of the PROSPECT study

### Design

The study is a prospective, open-label, randomised, multicenter and group-sequential phase IIb study in patients with locally advanced or metastasized pancreatic cancer who are treated with a palliative chemotherapy (stratified for risk) using either gemcitabine alone or in combination with cisplatin, 5-fluorouracil (5-FU) and folinic acid, both with or without enoxaparin. It was registered with isrctn.org (CCT-NAPN-16752) and controlled-trials.com (ISRCTN02140505). Ethical approval was obtained of the ethics committee of the Charite University Clinic Berlin.

**Figure 2:**
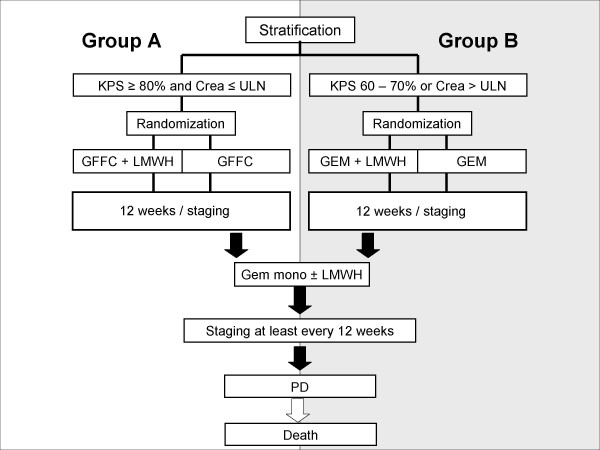
**Study design**. Figure legend text: KPS, Karnowsky Performance Status; Crea, Creatinine; ULN, upper limit of normal (> 1.1 mg/dl for men and > 0.9 mg/dl for women); GFFC, combination of gemcitabine, 5-fluorouracil, folinic acid and cisplatin; GEM, gemcitabine monotherapy; PD, partial remission; CR, complete remission.

Primary stratification takes place according to Karnofsky performance status (KPS) and kidney function. Patients with a KPS > 80% and normal kidney function (creatinine plasma level < upper limit of normal) receive GFFC therapy (gemcitabine 1 g/m^2 ^(30 min), 5-FU 750 mg/m^2 ^(24 h), and folinic acid 200 mg/m^2 ^(30 min), cisplatin 30 mg/m^2 ^(90 min)day 1, 8; q3w). After 12 weeks of initial GFFC chemotherapy all patients with no cancer progression receive standard therapy (gemcitabine 1 g/m^2 ^(30 min), d1, 8, 15; q4w). Patients with KPS < 80% and/or increased creatinine plasma levels start with the standard therapy (gemcitabine 1 g/m^2 ^(30 min), day 1, 8, 15; q4w). Both treatment groups were randomized to enoxaparin (1 mg/kg BW qd for the first three months followed by 40 mg qd for an additional three months) or to no anticoagulation. For further details see figure 2.

### Endpoints

The primary endpoint is the reduction of symptomatic clinically relevant venous thromboembolic events in the group treated with enoxaparin compared to no treatment arm within 3 month. Clinically relevant thromboembolic events are defined as symptomatic DVT of the leg and/or pelvis and/or pulmonary embolism.

Secondary endpoints include: 1) Incidence of symptomatic thromboembolic events within the first 6, 9 and 12 months; 2) Incidence of asymptomatic, subclinical deep vein thrombosis during months 6, 9 and 12 determined by compression ultrasound sonography; 3) Total survival, rates of remission at 3, 6, 9 and 12 months, toxicity of the therapeutic regimen, time to tumour regression and quality of life during chemotherapy with or without enoxaparin and the rate of major bleeding. Overall survival and time to tumour progression will be analysed separately following intention to treat and according to protocol principles. A subgroup analysis of patients without any thromboembolic events during the study will be performed to explore possible pleitropic effects.

### Study population

Inclusion criteria were the following: 1) Histologic or cytologic confirmed pancreatic cancer stage IV; 2) No previous radio- or chemotherapy of the primary tumour or the reference lesions; 3) KPS ≥ 60%; 4) Measurable tumour lesion confirmed by computed tomography (CT) or magnetic resonance tomography (MRT) within the last 14 days; 5) No DVT within the last 2 years, adequately compliant patient and home residence within geographical proximity to the hospital which allows an adequate follow-up; 6) Sufficient bone marrow function (leukocytes ≥ 3.5 × 10^9^/l, thrombocytes ≥ 100 × 10^9^/l); 7) Written informed consent; 8) Age ≥ 18 years, male and female patients in childbearing age have to have an adequate contraception during and up to 6 months after the study.

Exclusion criteria included the following: 1) Acute infection or pre-existing indication for anticoagulation. Bleeding within the last 2 weeks or increased risk of bleeding (severe impairment of coagulation, active stomach-gut ulcers or surgery within the last 2 weeks); 2) Body weight < 45 or > 100 kg; 3) Pregnancy/lactation or insufficient contraception during study; 4) Psychiatric disease; 5) Severe concomitant disease that is not compatible with a participation in the study; 6) Hypersensitivity against one of the drugs used or against structurally similar drugs; 7) Patients with severely impaired renal function (creatinine clearance < 30 ml/min).

### Investigational plan

**Figure 3:**
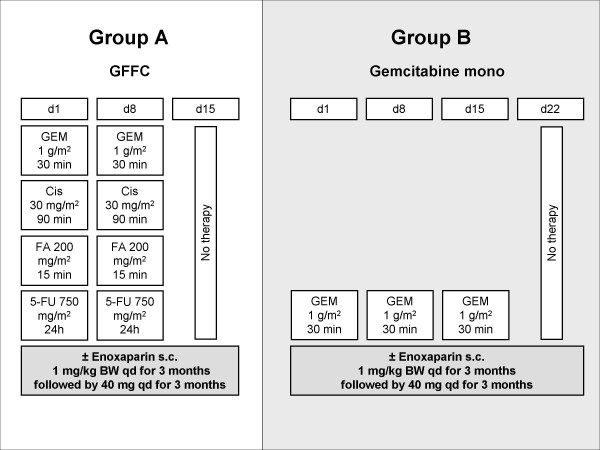
**Treatment pathway**. Figure legend text: GFFC, combination of gemcitabine, 5-fluorouracil, folinic acid and cisplatin; GEM, gemcitabine; CIS, cisplatin; FA, folinic acid; 5-FU, 5 fluorouracil; d, day.

Therapy is continued until documented progression is observed or as long the patient benefits from therapy. CT or MRT is performed at least every 12 weeks or earlier in case of suspected progression (figure 3).

*Dose adjustment *for enoxaparin is recommended in patients with impaired kidney function and thrombocytopenia. Following the NCI-CTC (National Cancer Institute Common Toxicity Criteria) a reduction of enoxaparin to 0.5 mg/kg is recommended in NCI stage II (thrombocytes 50.000 – 75.000/μl) until an increase in thrombocytes > 75.000/μl. Enoxaparin is to be interrupted in thrombocytopenia below 50.000/μl (NCI III).

To treat *nausea and vomiting *during the study, patients in the GFFC group receive preventively tropisetrone (5 mg) and dexamethasone (12 mg) for example and if necessary additionally alizapride (100 mg) on days 0, 1, 2, 8, 9 and 10. For patients allocated to standard gemcitabine therapy preventive alizapride 100 mg and dexamethasone 8 mg were recommended. In case of any *toxicity *(> NCI grade II) the dose is adjusted according to the protocol guidelines. *Concomitant therapy: *It is recommended for patients with a haemoglobin level below 10.5 mg/dl to give 150 μg/week darbopoetin alfa. Enteral or parenteral additional nutrition, adequate pain-relief (NCI-scheme [37]) and adequate vitamin supplementation is provided.

### Statistical assumptions and timelines

A risk of symptomatic thromboembolism of 10% and an absolute risk reduction of 7% under enoxaparin treatment to 3% are assumed within the first 3 months. Enrolment of 540 patients will provide an 80% power to verify this difference.

After amendment 1 in June 2006, all patients without symptomatic event of DVT or PE were to receive an ultrasound-based diagnostic of the legs every three months to detect subclinical thrombosis. All subclinical or clinical events of DVT/PE are documented centrally. The significance of the events are evaluated by an independent blinded "event review board". All patients will be evaluated regarding the "intent to treat" and the "according to protocol" analysis.

### Interim analysis

According to protocol, an interim analysis for safety was conducted when 152 patients were enrolled into the PROSPECT trial. There was no difference in overall bleeding as well as major bleedings between the two groups (5 pts vs. 6 pts, Chi^2^: 0.763). No heparin-induced thrombocytopenia was observed.

## Discussion

Besides the PROSPECT study, there are currently only three registered ongoing trials investigating the role of LMWH in patients with pancreatic cancer (NCT00426127, NCT00031837 and NCT00312013) all registered at clinicaltrials.gov the official NIH/FDA website for study registration. *NCT00426127 *is a small non-randomized, uncontrolled, open label efficacy study in 27 patients with advanced pancreatic cancer. Docetaxel and liposomal doxorubicin chemotherapy is administered together with enoxaparin. Primary outcome is tumour response measured by CT scans after cycles 3 and 6. Secondary outcome is incidence of elevated D-Dimer measured by drawing D-Dimer levels every cycle. Safety and effect of chemotherapy regimen on D-Dimer measured by drawing D-Dimer levels every cycle are also investigated.

*NCT00031837 *is a randomized, multicentric active controlled study comparing dalteparin to placebo in 400 patients with histologically or cytologically confirmed pancreatic adenocarcinoma or poorly differentiated carcinoma of the pancreas considered ineligible for curative resection (unresectable or metastatic). Chemotherapy regimen used is gemcitabine monotherapy. Primary or secondary endpoints have not been disclosed. Recruitment started in October 2002 (first record received at clinicaltrials.gov). The trial is at present still active but not enrolling (last update October 2008). *NCT00312013 *is *a *randomized, open-label, uncontrolled, parallel assignment safety/efficacy study in 500 patients with advanced malignancies (including pancreatic cancer). Effects of nadroparin in patients with lung, pancreas or prostate cancer are investigated. Primary outcomes are death from causes at study end (follow-up until at least 46 weeks after randomization). Secondary outcome is time to tumour progression. Recruitment started in March 2006 and is still active but not recruiting (last update November 2008).

### Rationale for the combination of gemcitabine, cisplatin, 5-fluorouracil and folic acid

Most randomized trials of the last decade failed to demonstrate a significant benefit from gemcitabine based combination therapies for pancreatic cancer. But sub-analyses showed a survival benefit for more intensive therapies in patients with good Karnofsky Performance Status [38,39]. Consistent with these results, we implemented a primary stratification regarding to medical constitution. Data from several phase II and randomised phase III studies, which investigated a combination of gemcitabine and cisplatin vs. gemcitabine monotherapy, suggested an increased response of tumours to therapy (between 11 and 35%) and a prolonged median survival (between 5.7 and 8.4 months) reaching significance in patients with good performance status [38,40-47]. Pre-clinical data document further the synergism between gemcitabine and 5-FU, since both inhibit the de-novo synthesis of thymidinribonucleotides [48]. The combination of gemcitabine and 5-FU has furthermore been investigated in clinical trials, amongst these data of our own group [49,50]. In a phase I study we were able to demonstrate, that a combination of gemcitabine (100 mg/m^2^), 5-FU (750 mg/m^2^) and folinic acid (200 mg/m^2^) is an effective therapeutic regimen with a low number of side effects resulting in remissions and a median survival of more than 8 months in outpatients [50]. In a recently published phase II study, El-Rayes and colleagues treated 47 patients with advanced or metastasised pancreatic cancer with a combination of gemcitabine, cisplatin and 5-FU. Excellent tumour response (26%) was achieved and the 1-year survival rate for patients with metastases was 34% and the median overall survival 8.6 months [51].

Actually, in a wide range of completed phase III studies, there are only two trials presenting a significance improvement in overall survival in patients with advanced or metastatic pancreatic carcinoma. One of them used the combination of gemcitabine with capecitabine (5-FU prodrug) [52] the other highlights the superiority of gemcitabin in addition with erlotinib over single agent gemcitabine [53]. The clinical impact of these studies is now under discussion.

### Rationale for the choice of the anticoagulant

Recently, heparin and especially LMWH have been shown to inhibit tumour growth and to increase the efficacy of chemotherapy [54,55]. In comparison to unfractionated heparin (UFH), LMWH has greater inhibitory activity against growth factors, angiogenesis, and coagulation activity [56]. In addition to the experimental tumour models reporting tumour growth-inhibiting activity of LMWH, there are some substantial tumour responses with LMWH in a variety of cancers [31,32,57]. Along with the endothelial cell-damaging effects of cisplatinum, the antiproliferative effect of heparins on endothelial cells could be another explanation for the antitumour activity of the current treatment regimen [58-60]. Finally enoxaparin is recommended by the recently published IUA guidelines for the prolonged prevention of thrombosis [36]. An interims analysis of this pilot trial showed that enoxaparin use (1 mg/kg BW for the first 3 months followed by 40 mg for additional 3 months) was not associated with an increase in overall and major bleeding as compared to observation and recruitment has been resumed. This lack of increased bleeding with LMWH in this patient population treated with chemotherapy has also been observed when compared to vitamin K antagonists [23,24].

## Conclusion

PROSPECT is a pivotal study in elucidating the role of low molecular weight heparins in advanced pancreatic cancer. Its results will lead to a new understanding of the role of heparins in the prevention of venous thromboembolism and of their effect on survival, remission rates and toxicity of chemotherapeutic regimens.

## Competing interests

PROSPECT is sponsored by a unrestricted grant of Sanofi-Aventis Deutschland GmbH, Berlin, Germany to Prof. Dr. Hanno Riess. DKB is an employee of Sanofi Aventis.

## Authors' contributions

HR and HO are the principal investigators of the study. Together with AH and UP they have written the outline and protocol of the study. HR, UP, AH, JS, BO, TS, BD and HO have been active recruiting patients into the study. DKB and PB have drafted the manuscript. All authors read and approved the final manuscript.

## Pre-publication history

The pre-publication history for this paper can be accessed here:


